# Prevalence and characteristics of *pks* gene cluster harbouring *Klebsiella pneumoniae* from bloodstream infection in China

**DOI:** 10.1017/S0950268820000655

**Published:** 2020-03-12

**Authors:** Qiucheng Shi, Jingjing Quan, Peng Lan, Danyan Huang, Jiancang Zhou, Yan Jiang, Yunsong Yu

**Affiliations:** 1 Department of Infectious Diseases, Sir Run Run Shaw Hospital, Zhejiang University School of Medicine, Hangzhou, China; 2 Key Laboratory of Microbial Technology and Bioinformatics of Zhejiang Province, Hangzhou, China; 3 Department of Clinical Laboratory, Anhui Province Hospital, Hefei, China; 4 Department of Critical Care Medicine, Sir Run Run Shaw Hospital, Zhejiang University School of Medicine, Hangzhou, China

**Keywords:** Bloodstream infection, colibactin, genotoxic *Klebsiella pneumoniae*, hypervirulent *Klebsiella pneumoniae*, *pks* gene cluster

## Abstract

Bloodstream infection (BSI), caused by *Klebsiella pneumoniae*, is associated with high morbidity and mortality, where the *pks* gene cluster plays a major role in their occurrence and prevalence. Information on the prevalence and characteristics of this gene cluster in *K. pneumoniae* is currently limited in mainland China. We therefore undertook a multicentre longitudinal study which revealed the prevalence, overall, community-onset and hospital-acquired BSI to be 20.5%, 28.3% and 13.0%, respectively. Compared to *pks*-negative, *pks*-positive isolates were significantly more susceptible to antimicrobial agents with a low incidence (5.1%) of multidrug-resistance and with infrequent extended-spectrum beta-lactamase (ESBL) production. Among *pks*-positive isolates, ST23 (78/117) and ST65 (20/117) were the dominant sequence types, and the majority harboured virulence genes. Community-onset BSI patients infected with *pks*-positive isolates had a higher proportion of liver abscesses and a lower proportion of biliary obstructions (*P* < 0.05). The *pks*-positive isolates were mostly sporadic in the phylogenetic tree, with a 65.8 and 47.0 average allele difference between Clade 1 and Clade 2, respectively. We concluded that although *pks*-positive *K. pneumoniae* were generally susceptible to antimicrobials, the high prevalence of such isolates in community cases and the genotoxicity, merits further investigation.

## Introduction

Bloodstream infection (BSI) is associated with high morbidity and mortality rates. According to recent data from the China Antimicrobial Surveillance Network (CHINET), *Klebsiella pneumoniae* accounted for 15.4% of BSIs, and a mortality rate of 54.3% [1, 2]. The virulence of *K. pneumoniae* in the bloodstream is enhanced by its *pks* gene cluster, which is a hybrid non-ribosomal peptide synthetase-polyketide synthase assembly line that represents 19 genes (*clb*A to *clb*S) [3]. This gene cluster is responsible for colibactin synthesis and was first discovered in the extraintestinal pathogenic *Escherichia coli* strain IHE3034 [4]. The cluster is associated with DNA double-strand breaking and chromosome aberrations, which leads to senescence of epithelial cells and apoptosis of immune cells [4]. The presence of the *pks* gene cluster in *E. coli* has also been linked to bacteraemia, meningitis, and in patients with colorectal cancer [5, 6].

The *pks* genes, *clb*B and *clb*N, are significantly associated with BSIs and were found to be present in 58% of *E. coli* group B2 related bacteraemia isolates [7]. Moreover, bacterial loads of *K. pneumoniae* in the bloodstream in an experimental meningitis model in mice were shown to decline in those infected with *pks* gene (*clb*A) knockout isolates [8]. The *pks* gene cluster is globally diverse, but was found to be relatively infrequent (3.5%) among *K. pneumoniae* clinical isolates in Europe [9]. However, the gene cluster was recently detected in 17% of bloodstream-sourced *K. pneumoniae* isolates from the Taiwan region and also related to serotype K1, the most common serotype of hypervirulent *K. pneumoniae* (hvKP) in Asia [10, 11].

Data on the prevalence of the *pks* gene cluster in mainland China are limited. The current study therefore aimed to investigate its prevalence in bloodstream-sourced *K. pneumoniae* clinical isolates from a national multicentre longitudinal program in China. Microbiological, molecular and clonality characterisation of the *pks* gene cluster in a large collection of isolates was undertaken.

## Materials and methods

### Bacterial isolates collection

Five hundred and seventy-one *K. pneumoniae* isolates were selected from our previous 15 monthsstudy of extended-spectrum beta-lactamase (ESBL) production of *E. coli* and *K. pneumoniae* BSIs in mainland China [12]. Samples were included from 28 tertiary hospitals in 22 provinces and municipalities, which covered about one billion people. Community-onset BSIs were defined as infections which occurred in non-hospitalised patients, or less than 48 h after admission to hospital [13]; 279 isolates were defined as community-onset *K. pneumoniae* (COK), and 292 as hospital-acquired *K. pneumoniae* (HAK). Isolates were identified by matrix-assisted laser desorption ionisation-time of flight mass spectrometry (Bruker Daltonics, Bremen, Germany). The study was approved by our hospital ethics committee (20130910-13) with a waiver of informed consent.

### Detection of the *pks* gene cluster in *K. pneumoniae* isolates

The presence of *pks* genes (*clb*A, *clb*B, *clb*N and *clb*Q) among all isolates was screened for by the conventional polymerase chain reaction (Takara, Dalian, China) [4] using specific primers and parameters as listed in Table S1.

### Antimicrobial susceptibility testing

Antimicrobial susceptibility testing of 12 antimicrobial agents against all isolates was performed using an agar microdilution method and results were interpreted according to the Clinical & Laboratory Standards Institute [14]. Susceptibility to tigecycline, however, was determined using broth microdilution with cation-adjusted Mueller-Hinton broth, and interpreted according to the European Committee on Antimicrobial Susceptibility Testing breakpoint [15].

### Whole-genome DNA sequencing and analysis

Total DNA was extracted from 117 *pks*-positive *K. pneumoniae* isolates (QIAGEN, Hilden, Germany) and subjected to whole genome sequencing with 2 × 150 bp paired-end reads (Illumina HiSeq X Ten, San Diego, California, USA). The derived short reads were assembled into contigs in a CLC Genomics Workbench 9.5.1 (QIAGEN, Germany) by automatic word size and bubble size, with a minimum contig length of 200 base pair. The genome sequence was submitted to the European Nucleotide Archive (accession number PRJEB32094). Multilocus sequence types (MLST) were identified by mapping the assembled contigs against the *K. pneumoniae* MLST database on the Center for Genomic Epidemiology (CGE) server [16]. The assembled contigs were also used to screen for acquired antimicrobial resistance genes by ResFinder 2.1 on the CGE server [17], with 90% identity and 60% minimum length. Virulence genes and *wzi* alleles were identified with reference to the Pasteur Institute website (https://bigsdb.pasteur.fr/klebsiella/klebsiella.html).

### Clonality analysis by core-genome multilocus sequence typing (cgMLST)

FASTA files of each isolate were imported into SeqSphere + 4.1.9 (Ridom GmbH, Münster, Germany) for stable cgMLST analysis to identify cluster types (CT) with default parameters. *K. pneumoniae* NTUH-K2044 (GenBank accession no. NC_012731.1) was used as a reference with a standard set of 2358 genes for gene-by-gene comparison, and the cgMLST comparison table was established. SeqSphere + 4.1.9 was also utilised to obtain neighbour-joining (N-J) and minimum span trees. The genome sequence used for the construction of phylogenetic trees was searched on the National Center for Biotechnology Information (NCBI) database; the isolate identifier and location origin are listed in Table S2.

### Statistical analysis

For categorical variables and continuous variables, comparisons were performed by the Fisher's exact test and *t*-test. A *P*-value < 0.05 was considered statistically significant. The statistical software used was Prism5 (Graph Pad Software, California, USA).

## Results

In total, 117 *pks* gene cluster positive representatives were identified among 571 *K. pneumoniae* isolates; 79 were classified as COK (28.3%) and 38 (13.0%) as HAK isolates (*P* < 0.0001).

### Antimicrobial susceptibility of isolates

As compared with *pks*-negative *K. pneumoniae*, *pks*-positive isolates were significantly more susceptible to 12 antimicrobial agents (Table 1), including, *β*-lactams, *β*-lactam/*β*-lactamase inhibitors, fluoroquinolones, cephamycin, aminoglycosides and oxacephem, except for tigecycline. For instance, the susceptibility rate of cefepime, ceftazidime, ciprofloxacin and amoxicillin/clavulanate was 91.5%, 92.3%, 94.0% and 90.6% in *pks*-positive isolates, respectively, compared with *pks*-negative isolates, where the respective rate for these antibiotics was 75.1%, 74.0%, 74.0% and 58.8%. All *pks*-positive isolates were susceptible to carbapenem agents while over 90% of their *pks-*counterparts showed susceptibility. Multidrug resistant (MDR) was defined as acquired resistance to at least one agent in three or more antimicrobial classes, and 5.1% (6/117) of *pks*-positive isolates and 28.6% (130/454) of *pks*-negative isolates were MDR.

**Table 1.** Susceptibility of *pks*-positive and *pks*-negative *K. pneumoniae* isolates to antimicrobials

Antibiotics (*n*, %)	*pks*-Positive (*n* = 117)	*pks*-Negative (*n* = 454)	*P* value
Amikacin	116 (99.1)	411 (90.5)	**0**.**0011**
Cefepime	107 (91.5)	341 (75.1)	**<0**.**0001**
Cefoxitin	107 (91.5)	263 (58.0)	**0**.**0003**
Ceftazidime	108 (92.3)	336 (74.0)	**<0**.**0001**
Cefuroxime	99 (84.6)	263 (58.0)	**<0**.**0001**
Ciprofloxacin	110 (94.0)	336 (74.0)	**<0**.**0001**
Imipenem	117 (100.0)	416 (91.6)	**0**.**0010**
Meropenem	117 (100.0)	414 (91.2)	**0**.**0004**
Moxalactam	117 (100.0)	413 (90.1)	**0**.**0010**
Tigecycline	112 (95.7)	421 (92.7)	0.3023
Amoxicillin/clavulanate	106 (90.6)	267 (58.8)	**<0**.**0001**
Cefoperazone/sulbactam	109 (93.1)	345 (76.0)	**<0**.**0001**
Piperacillin/tazobactam	114 (97.4)	350 (77.1)	**<0**.**0001**

### Clinical characteristics of community-onset infection patients

Clinical outcome data were available for 249 of the 279 patients classified as community-onset BSI. Table 2 shows that patients infected with *pks*-positive *K. pneumoniae* were significantly younger than those with *pks*-negative isolates (*P* < 0.05). Likewise, a much higher proportion of *pks*-positive patients presented with a liver abscess (*P* < 0.05), but were less likely to have biliary obstruction (*P* < 0.05). There was no significant difference in clinical prognosis and outcomes between patients harbouring *pks*-positive or *pks*-negative isolates (*P* > 0.05).

**Table 2.** Demographic and clinical data of patients with community-onset BSI according to isolation of *pks*-positive and *pks*-negative *K. pneumoniae*

Community-onset BSIs patients	*pks*-Positive (*n* = 73)	*pks*-Negative (*n* = 176)	*P* value
Age (mean, IQR)	56.8 (48–65)	61.9 (51.75–75)	**0**.**0185**
Male (*n*, %)	50 (68.5)	109 (62.0)	0.3852
Diagnosis (*n*, %)			
Urinary tract infection	2 (2.7)	12 (6.8)	0.2446
Biliary tract infection	6 (8.2)	25 (14.2)	0.2141
Pneumonia	7 (9.6)	17 (9.7)	>0.9999
Liver abscess	24 (32.9)	27 (15.3)	**0**.**0031**
Skin and soft tissue infection	1 (1.4)	2 (1.1)	>0.9999
Primary sepsis	31 (42.5)	79 (44.9)	0.7800
Others	2 (2.7)	14 (7.9)	0.1615
Underlying disorders (*n*, %)			
Diabetes	30 (41.1)	68 (38.6)	0.7761
Prostatic hyperplasia	1 (1.4)	12 (6.8)	0.1160
Biliary obstruction	11 (15.1)	50 (28.4)	**0**.**0346**
Hepatic cirrhosis	5 (6.8)	13 (7.4)	>0.9999
Chronic renal failure	6 (8.2)	10 (5.7)	0.5703
Heart failure	2 (2.7)	8 (4.5)	0.7279
Treatment outcome (*n*, %)			
Curing	27 (37.0)	55 (31.3)	0.3792
Improvement	39 (53.4)	102 (58.0)	0.5747
Failure	7 (9.6)	16 (9.1)	>0.9999
Relapse	0 (0)	3 (1.7)	0.5578

### Molecular characteristics of *pks*-positive *K. pneumoniae*

ST23 (78/117) and ST65 (20/177) were the dominant sequence types identified in *pks*-positive isolates and were similarly distributed in COK and HAK patients (*P* > 0.05); eight other STs (ST380 (6), ST268 (3), ST133 (3), ST1660 (2), ST2058 (1), ST2846 (1), ST1265 (1) and ST3 (1)) accounted for 15.4% of isolates, and one isolate had a novel ST (Table 3). Genome sequencing showed that K1 (81/117) and K2 (25/117) were the predominant serotypes among *pks*-positive isolates. Six isolates fell into other K serotypes (K3 (1), K34 (2) and K20 (3)), and five were serologically un-typeable. Virulence genes were ubiquitous in *pks*-positive isolates, particularly the siderophore encoding genes *iro*N (salmochelin), *iuc*A (aerobactin) and *ybt*A (yersiniabactin) which were found in frequencies of 88.0%, 90.6% and 100% respectively. Moreover, 72.6% of all isolates were *rmp*A positive, the positive regulator of the mucoid phenotype [18]. There was no significant difference in the distribution rate of K serotype and virulence genes between *pks*-positive COK and HAK (Table 3). As for drug resistance determinants, no isolate proved positive for carbapenemase genes, and only seven isolates representative of the CTX-M-1 group and six of the CTX-M-9 group were identified (Table 3). Likewise, we selected 23 *pks*-negative *K. pneumoniae* from this project, which fell into 19 different STs and 20 different K serotypes (*wzi* alleles), that indicated the diversity of *pks*-negative isolates from BSI. The screen of virulence genes showed the frequencies of *rmpA*, *iro*N, *iuc*A and *ybt*A to be 21.7%, 39.1%, 34.8% and 39.1%, respectively. There were 56.5% (13/23) of isolates producing ESBLs (CTX-M-1 group (4), CTX-M-9 group (6) and SHV group (3)) and three isolates were *bla*_KPC_ positive.

**Table 3.** MLST, K serotype, virulence genes and drug resistance genes, of *pks*-positive isolates of *K. pneumoniae.*

Characteristics (*n*, %)	*pks*-Positive	COK (*n* = 79)	HAK (*n* = 38)	*P* value
MLST				
ST23	78	54 (68.4)	24 (63.1)	0.6760
ST65	20	13 (16.5)	7 (18.4)	0.7975
Others	18	11 (13.9)	7 (18.4)	0.5878
Unknown	1	1 (1.3) ^Δ^	0 (0)	>0.9999
K serotype				
K1	81	58 (73.4)	23 (60.5)	0.1999
K2	25	16 (20.3)	9 (23.7)	0.8100
Others	6	3 (3.8)	3 (7.9)	0.3884
Not defined	5	2 (2.5)	3 (7.9)	0.3274
Virulence genes				
*iroN*	103	73 (92.4)	30 (78.9)	0.0638
*iucA*	106	73 (92.4)	33 (86.8)	0.3330
*rmpA*	85	58 (73.4)	27 (71.1)	0.8266
*ybtA*	117	79 (100.0)	38 (100.0)	NA
*mrkD*	115	77 (97.5)	38 (100.0)	>0.9999
*allS*	81	57 (72.2)	24 (63.2)	0.3932
Drug resistance genes (ESBL)				
*bla*_CTX−M−55_	3	1 (1.3)	2 (5.2)	0.2459
*bla*_CTX−M−15_	2	2 (2.5)	0 (0)	>0.9999
*bla*_CTX−M−3_	2	0 (0)	2 (5.2)	0.1036
*bla*_CTX−M−14_	5	3 (3.8)	2 (5.2)	0.6592
*bla*_CTX−M−104_	1	1 (1.3)	0 (0)	>0.9999
*bla*_SFO−1_	1	0 (0)	1 (2.6)	0.3248

^∆^ ST of COK15 *phoE*: 28 (A53G)

### Clonality of *pks*-positive isolates

**Fig. 1.**
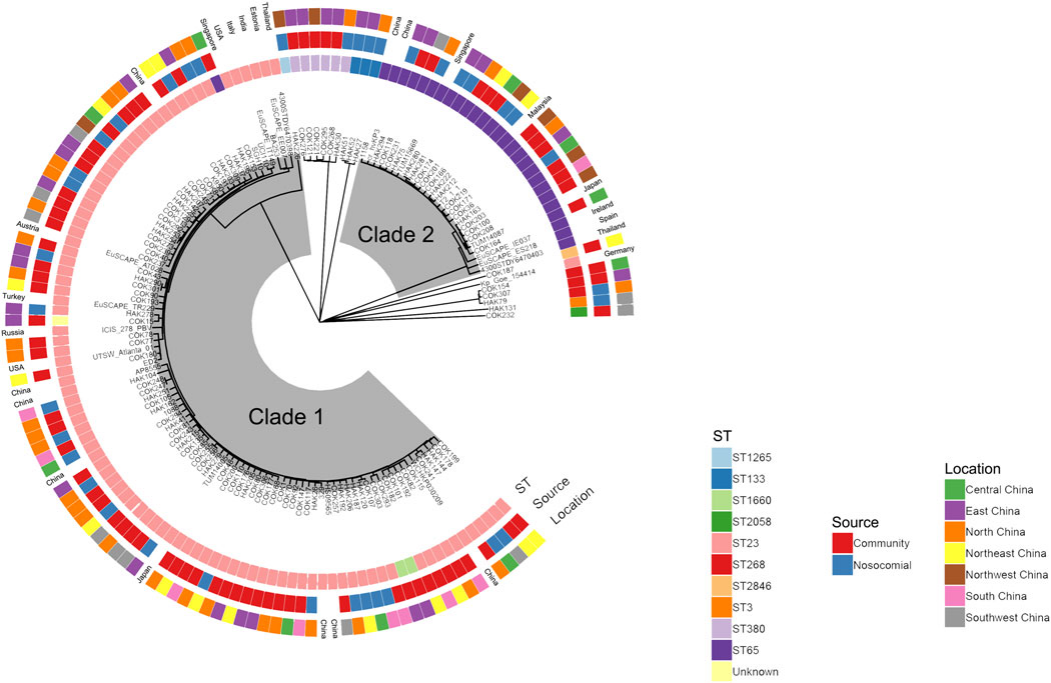
The N-J tree of *pks*-positive isolates (isolates in the current study and the genome from the NCBI database) was constructed. Sequence types, source and geographic location were shown.

To investigate clonal linkage of the *pks*-positive isolates, an N-J tree was constructed based on the cgMLST allelic profiles with default parameters. Except for two unidentified isolates, 115 isolates and 27 genome sequences from the NCBI database were divided into nine clades (allelic differences >1000). The two major clades (Clade 1 and Clade 2) mainly consisted of ST23 and ST65 clone groups, respectively (Fig. 1). The results suggested that either the source, or the geographical origin of *pks*-positive isolates was mixed in the phylogenetic tree, and indicated that community and hospital isolates were phylogenetically related, and no specific epidemic clone existed in mainland China. Moreover, by comparison with genomes from the worldwide database, ST23 isolates from Russia, Japan, USA, Austria, Singapore and Italy were all related to *pks*-positive ST23 isolates in Clade 1. Similar results were found for ST65 isolates, with those from Singapore, Malaysia, Japan, Ireland and Spain being closely similar to representatives of this ST from China, and demonstrate the sporadic presence of *pks*-positive *K. pneumoniae* worldwide.

To further explore the differences of the isolates from this study, the allelic distance of Clade 1 and Clade 2 was calculated. In Clade 1, after excluding HAK226 with an average distance of 318.4.4 alleles, there were 80 isolates with an average distance of 65.8 alleles (Fig. S1A), and 95% of them were ST23; the remainder included ST1265 (1), ST65 (1), ST1660 (2) and one unknown ST. Two paired isolates that showed no allelic difference were COK301/HAK290 (CT1895, Jiaxing) and COK247/COK248 (CT1876, Hohhot). COK255/COK256 (CT1869, Guiyang) and HAK253/HAK254 (CT1907, Hohhot) differed by one allele while COK107/HAK120 (CT1847, Guangzhou) had five allelic differences. The average distance was 47.0 alleles in Clade 2, and all of 19 isolates were ST65 with different CT, except that HAK280/HAK281 (CT1915, Fuzhou) had no allelic difference from isolates in the same hospital (Fig. S1B). Six paired *pks*-positive isolates from the same city showed less than 10 allelic differences.

## Discussion

BSI caused by *K. pneumoniae* is associated with high morbidity and mortality, where the *pks* gene cluster plays a major role in the occurrence and prevalence of BSI [8]. Previous studies from the region have reported the prevalence of the *pks* gene cluster in *K. pneumoniae* to be 17% in Taiwan [10] and 26.8% in Changsha, China [19]. The current study extends these data to encompass a large number of BSI *K. pneumoniae* isolates from 28 hospitals in 22 provinces and municipalities in mainland China and reports an overall prevalence of 20.5%.

Bloodstream-sourced *pks*-positive isolates share many microbiological and clinical characteristics associated with the hvKP variant which first emerged in Taiwan in the mid 1980s [11]. Infection with such strains was most often characterised by community-onset, and relatively infrequent resistance to antibiotics [20]. In our study, 67.5% of *pks*-positive isolates were community-onset, which was twice that of the hospital-acquired infections, and were also more susceptible to antimicrobials, as reported by Chen *et al*. [10]. Moreover, previous studies have found that hvKP strains isolated from four different continents were almost exclusively of serotype K1 (93%), or K2 [21], and ST23 and ST65 were predominant in such strains [22]. The great majority (90.6%) of our *pks*-positive isolates were K1 or K2 serotype, and ST23 and ST65 accounted for 82% of the study sample. The *pks* gene cluster was shown to co-exist with the *ybt* locus and T4SS-*mob*BC on integrative conjugational elements *Kp*10 (ICE*Kp*10) in the vast majority of the ST23 clonal group [23], which explains the presence of *ybtA* in all our *pks*-positive isolates.

There is no universal clinical definition of hvKP infections but it is generally agreed that they are most commonly isolated from patients with community-associated liver abscesses, metastatic meningitis and endophthalmitis, and that such cases usually have normal biliary and hepatic function [24]. In this study, patients infected with *pks*-positive *K. pneumoniae* had a higher proportion of liver abscesses and a lower rate of biliary disorders, which is consistent with the view that *pks*-positive isolates may play an important role in hvKP infections, as suggested by Lan *et al*. [22].

A recent study from South Korea reported that the *pks* gene cluster is a risk factor for 30-day mortality of *K. pneumoniae* BSI patients when accompanied MDR, but the relative MDR rates for *pks*-negative isolates is unclear [25]. In our study, 5.1% of *pks*-positive isolates were multidrug-resistant compared with 28.6% of *pks*-negatives. Although the MDR rate appears to be currently low in *pks*-positive isolates, active surveillance of these properties is warranted.

The threshold for the definition of strain relatedness within an outbreak cluster was set as a difference of <10 alleles, which would be considered a close relationship between strains [26]. Genome sequencing showed that the *pks*-positive isolates were mostly sporadic in the phylogenetic tree, with a 65.8 and 47.0 average allele difference between Clade 1 and Clade 2, respectively; these values indicate that there does not appear to be a widespread epidemic lineage of *pks*-positive *K. pneumoniae* nationwide in China. Indeed, only isolates from the same hospital showed a difference of <10 alleles, and these strains were found in both COK and HAK patients. However, two exceptions were noted for COK301/HAK290 and COK107/HAK120, where close clonality was evident among the community-onset and hospital-acquired isolates, which is suggestive of transmission of such lineages between these presentations. Apparent clonality of *E. coli* bacteraemia strains from community-acquired and healthcare-associated cases has been previously observed [27] and it is therefore possible that our general classification of some infections as COK or HAK, might have masked subtle differences in acquisition of the organism and mistakenly interpreted as close clonality of strains.

In conclusion, our study of 571 *K. pneumoniae* BSI isolates from patients in 28 tertiary hospitals in 22 provinces in mainland China, classified according to the presence or absence of the *pks* gene cluster, showed that the genetic lineages ST23 and ST65 predominated among *pks*-positive isolates. There was no evidence of a widespread epidemic clone and infections were mostly sporadic. Although *pks*-positive isolates were generally susceptible to antimicrobials, their higher prevalence in the community and the genotoxicity, represents a potential risk for treatment of BSI due to *K. pneumoniae.*

## Supplementary material

For supplementary material accompanying this paper visit https://doi.org/10.1017/S0950268820000655. click here to view supplementary material
