# Supplementary material for: Prevalence and characteristics of pks gene cluster harbouring Klebsiella pneumoniae from bloodstream infection in China
Source: Epidemiol Infect. 2020 Mar 12;148:e69. doi: 10.1017/S0950268820000655 (PMC7118716; doi:10.1017/S0950268820000655)
Supplement: Supplementary file 1 [file S0950268820000655sup.zip › S0950268820000655sup002.docx]

**Table S1** PCR primers used for screening *pks* gene cluster of *K. pneumoniae* isolates

| Target gene | Primers | Nucleotide sequence (5’-3’) | Tm (^o^C) | Reference |
| --- | --- | --- | --- | --- |
| *clbA* | clbAF | CTAGATTATCCGTGGCGATTC | 52 | [4] |
|  | clbAR | CAGATACACAGATACCATTCA | 49 |  |
| *clbB* | clbBF | GATTTGGATACTGGCGATAACCG | 55 | [4] |
|  | clbBR | CCATTTCCCGTTTGAGCACAC | 54 |  |
| *clbN* | clbNF | GTTTTGCTCGCCAGATAGTCATTC | 56 | [4] |
|  | clbNR | CAGTTCGGGTATGTGTGGAAGG | 57 |  |
| *clbQ* | clbQF | CTTGTATAGTTACACAACTATTTC | 49 | [4] |
|  | clbQR | TTATCCTGTTAGCTTTCGTTC | 49 |  |

All the reaction were performed in 30 repeats with 30 seconds for denaturation, 30 seconds for annealing, and 1minutes for elongation. The denaturation temperature was 95 ^o^C, and the elongation temperature was 72 ^o^C. The annealing temperature was adjusted by different primers. For *clbA*, *clbB*, *clbN*, and *clbQ*, the annealing temperature was 48, 52, 54, and 48 ^o^C, respectively.
