# Supplementary material for: Prevalence and characteristics of pks gene cluster harbouring Klebsiella pneumoniae from bloodstream infection in China
Source: Epidemiol Infect. 2020 Mar 12;148:e69. doi: 10.1017/S0950268820000655 (PMC7118716; doi:10.1017/S0950268820000655)
Supplement: Supplementary file 1 [file S0950268820000655sup.zip › S0950268820000655sup003.docx]

**Table S2** NCBI database isolates used for N-J tree.

| Isolate name | Geographic Location | ST |
| --- | --- | --- |
| 1158 | Taiwan, China | ST65 |
| 4300STDY6470403 | Thailand | ST65 |
| cr-hvkp3 | Beijing, China | ST65 |
| EuSCAPE_ES218 | Spain | ST65 |
| EuSCAPE_IE037 | Ireland | ST65 |
| T2-1-1 | Malaysia | ST65 |
| TUM14087 | Japan | ST65 |
| TUM15669 | Singapore | ST65 |
| 329 | Beijing, China | ST23 |
| 1088 | Zhejiang, China | ST23 |
| 4300STDY6470398 | Thailand | ST23 |
| AP8555 | Jiangxi, China | ST23 |
| BA253 | India | ST23 |
| ED2 | Taiwan, China | ST23 |
| EuSCAPE_AT028 | Austria | ST23 |
| EuSCAPE_EE007 | Estonia | ST23 |
| EuSCAPE_IT149 | Italy | ST23 |
| EuSCAPE_TR229 | Turkey | ST23 |
| HS09565 | Shanghai, China | ST23 |
| ICIS-278_PBV | Russia | ST23 |
| K950 | Jiangxi, China | ST23 |
| Kp_Goe_154414 | Germany | ST23 |
| SGH10 | Singapore | ST23 |
| TUM14005 | Japan | ST23 |
| WCHKP030209 | Sichuan, China | ST23 |
| UCI110 | USA | ST23 |
| UTSW_Atlanta_01 | USA | ST23 |
