# Supplementary figures and images for: Prevalence and characteristics of pks gene cluster harbouring Klebsiella pneumoniae from bloodstream infection in China
Source: Epidemiol Infect. 2020 Mar 12;148:e69. doi: 10.1017/S0950268820000655 (PMC7118716; doi:10.1017/S0950268820000655)

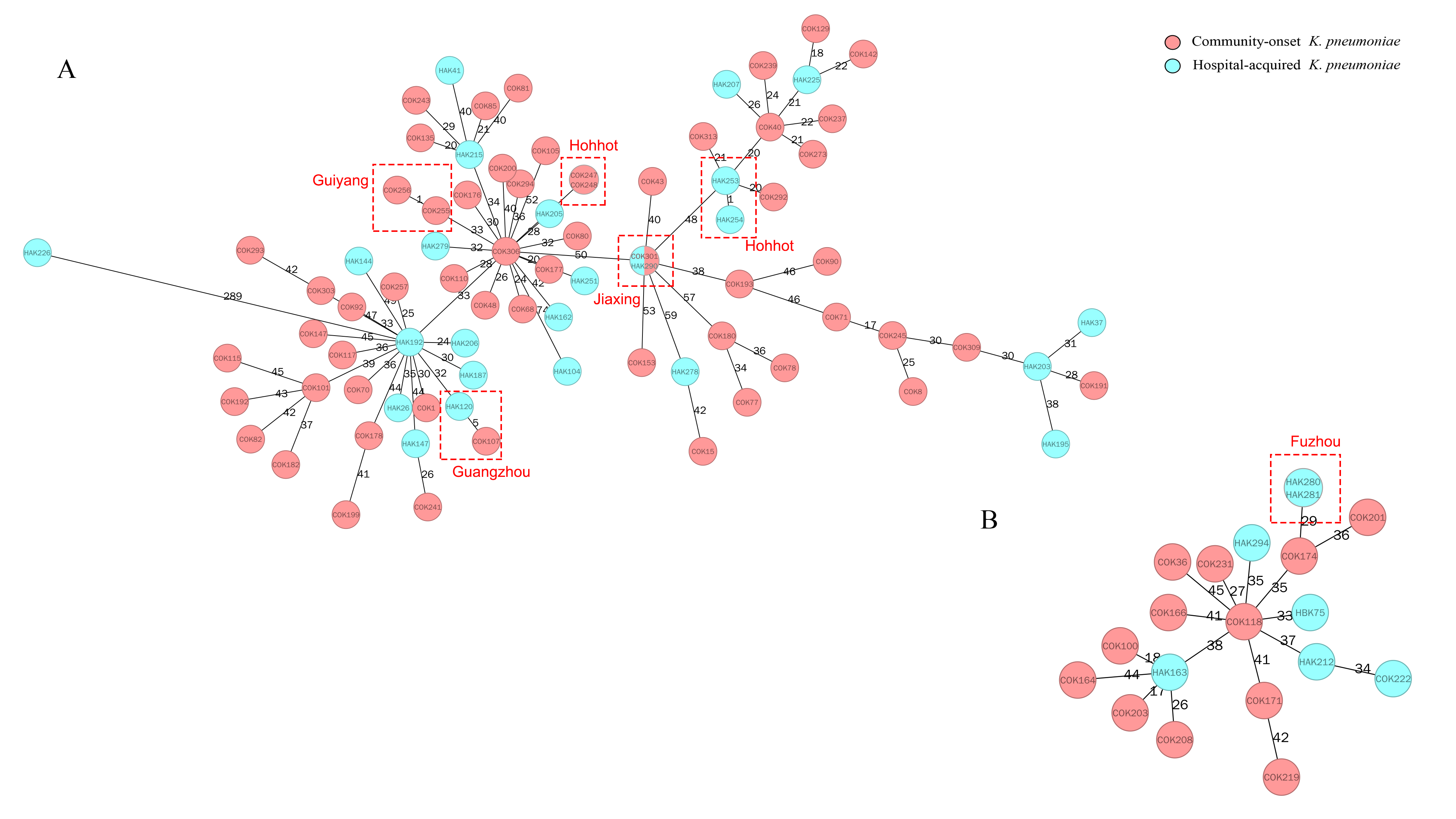

Supplement: Supplementary file 1 [file S0950268820000655sup.zip › S0950268820000655sup001.tif]
